# Identification of novel biomarkers related to neutrophilic inflammation in COPD

**DOI:** 10.3389/fimmu.2024.1410158

**Published:** 2024-05-30

**Authors:** Yuchen Huang, Yang Niu, Xuezhao Wang, Xiaochen Li, Yuanzhou He, Xiansheng Liu

**Affiliations:** ^1^ Department of Pulmonary and Critical Care Medicine, Tongji Hospital, Tongji Medical College, Huazhong University of Science and Technology, Wuhan, China; ^2^ Key Laboratory of Pulmonary Diseases of National Health Commission, Tongji Hospital, Tongji Medical College, Huazhong University of Sciences and Technology, Wuhan, China

**Keywords:** bioinformatics, biomarker, chronic obstructive pulmonary disease, Mendelian randomization, neutrophilic inflammation, single-cell RNA sequencing

## Abstract

**Background:**

Chronic obstructive pulmonary disease (COPD) is one of the most prevalent chronic respiratory diseases and the fourth cause of mortality globally. Neutrophilic inflammation has a vital role in the occurrence and progression of COPD. This study aimed to identify the novel hub genes involved in neutrophilic inflammation in COPD through bioinformatic prediction and experimental validation.

**Methods:**

Both the single-cell RNA sequencing (scRNA-seq) dataset (GSE173896) and the RNA sequencing (RNA-seq) dataset (GSE57148) were downloaded from the Gene Expression Omnibus (GEO) database. The Seurat package was used for quality control, dimensions reduction, and cell identification of scRNA-seq. The irGSEA package was used for scoring individual cells. The Monocle2 package was used for the trajectory analysis of neutrophils. The CIBERSORT algorithm was used for analysis of immune cell infiltration in the lungs of COPD patients and controls in RNA-seq dataset, and weighted gene co-expression network analysis (WGCNA) correlated gene modules with neutrophil infiltration. The Mendelian randomization (MR) analysis explored the causal relationship between feature DEGs and COPD. The protein–protein interaction (PPI) network of novel hub genes was constructed, and real-time quantitative polymerase chain reaction (qRT-PCR) was used to validate novel hub genes in clinical specimens.

**Results:**

In scRNA-seq, the gene sets upregulated in COPD samples were related to the neutrophilic inflammatory response and TNF-α activation of the NF-κB signaling pathway. In RNA-seq, immune infiltration analysis showed neutrophils were upregulated in COPD lung tissue. We combined data from differential and modular genes and identified 51 differential genes associated with neutrophilic inflammation. Using MR analysis, 6 genes were explored to be causally associated with COPD. Meanwhile, 11 hub genes were identified by PPI network analysis, and all of them were upregulated. qRT-PCR experiments validated 9 out of 11 genes in peripheral blood leukocytes of COPD patients. Furthermore, 5 genes negatively correlated with lung function in COPD patients. Finally, a network of transcription factors for NAMPT and PTGS2 was constructed.

**Conclusion:**

This study identified nine novel hub genes related to the neutrophilic inflammation in COPD, and two genes were risk factors of COPD, which may serve as potential biomarkers for the clinical severity of COPD.

## 1 Introduction

Chronic obstructive pulmonary disease (COPD) is the most common chronic respiratory disease characterized by persistent respiratory symptoms and airflow limitation accompanied by neutrophil infiltration in the airways and overproduction of mucus (1, 2). COPD is the fourth cause of mortality globally, imposing a heavy economic and social burden on the world (3). In China, a cross-sectional survey showed that the general prevalence of COPD, as defined by spirometry, was 8.6% in people aged 20 years and over and 13.7% in people aged 40 years and over. In 2019, about 212.3 million cases of COPD were recorded worldwide, with an age-standardized prevalence rate of 2,638.2 cases per 100,000 people. COPD led to 3.3 million deaths, with an age-standardized mortality rate of 42.5 per 100,000 population (4).

In COPD patients, neutrophil populations are increased by chemotactic mediators released from airway epithelial cells and macrophages in the lungs (5). However, the cellular and molecular variations within the systemic immune system of patients in COPD, particularly neutrophils, the most abundant immune cells in the circulation are poorly understood. Neutrophils are significant contributors to the development of COPD due to their production of cytokines, inflammatory mediators, oxygen-free radicals, and elastase (6). These substances cause inflammation in the airways, lung parenchyma, and pulmonary vasculature, destroying lung tissue (7). Furthermore, the number of neutrophils in COPD patients negatively correlates with lung function (8). Although neutrophils play a crucial role in COPD, the molecular mechanisms require further elucidation.

Recent applications of single-cell RNA sequencing (scRNA-seq) technologies have uncovered previously unknown molecular and functional diverse aspects of neutrophils, both in homeostasis and inflammatory disease (9). Notably, scRNA-seq studies on COPD have shown a significant underrepresentation of neutrophils. Therefore, we hypothesized that this innovative technique would allow us to delineate molecular and phenotypic variations in this crucial cell type in COPD and identify hub genes involved in neutrophil inflammation. These markers can act as proxies for disease progression to develop new treatment interventions.

Mendelian randomization (MR) is an epidemiological analysis method that utilizes genetic variation to identify causal associations between exposure factors and outcomes (10). Compared to traditional observational studies, MR is less susceptible to confounding and reverse causation. In this study, we further illustrated the causal role of hub genes in the risk of developing COPD by a two-sample MR approach.

## 2 Methods

### 2.1 Data source

The scRNA dataset (GSE173896) and the RNA-sequencing (RNA-seq) dataset (GSE57148) were obtained from GEO (http://www.ncbi.nlm.nih.gov/geo). The samples in both datasets were lung tissues from COPD and control subjects.

### 2.2 Analysis of single-cell sequencing data

Four COPD (GSM5282538, GSM5282539, GSM5282540, and GSM5282541) and three control nonsmokers (GSM5282546, GSM5282547, and GSM5282548) scRNA-seq lung tissue samples were obtained from GSE173896. The total number of cells in the COPD and control groups was approximately the same. Afterward, the Seurat package initially filtered scRNA-seq data to obtain higher-quality cells (11). For further analysis, cells and genes must fulfill the three quality control criteria: 1. The detected gene in at least three cells. 2. Detected at least 200 genes per cell. 3. No more than 15% of the genes are mitochondrial genes. With quality control, the overall count of cells in the COPD and control groups was 11,024 and 11,225, respectively. The canonical correlation analysis (CCA) algorithm integrated the seven samples and removed batch effects. Then, 3,000 highly variable genes were chosen for analysis, and the number of principal components (PCs) was programmed to 50 to get clusters of cells, which were visualized as a “UMAP” plot (12). All cells were subsequently labeled by the marker genes for each cell type. FindMarker was used to identify differential genes.

### 2.3 Trajectory analysis and gene set enrichment analysis

The Monocle2 was applied to perform single-cell trajectory analysis (13). All neutrophil objects were extracted, and the DDRTree method was used to reduce the dimension of cells. Then, the reduce Dimension function was implemented to determine the type of cell differentiation state. Finally, the “plot cell trajectory” function was employed to visualize the differentiation trajectory of cells.

Bioinformatics (https://www.bioinformatics.com.cn/) website was an open online platform for data processing and visualization. Gene Set Enrichment Analysis (GSEA) was used to identify enriched GO terms and KEGG associated with cell differentiation state. Sorting all of the differential genes according to logFC and GSEA was conducted on the website.

### 2.4 Identification of significantly related pathways in all cells

The level of gene set enrichment in each cell was assessed using the irGSEA package. This package was used to score individual cells and to generate numerous gene set enrichment score matrices. The half vlnplot and density scatterplot visualized some specific enriched pathways.

### 2.5 Immune infiltration analysis

We calculated the extent of immune cell infiltration in COPD and control lung tissue samples in GSE57148 by using the CIBERSORT algorithm. This algorithm set the PERM to 1000 and set the cutoff to p < 0.05 (14). The Pheatmap package created a heat map of different immune cells, and the Vioplot package displayed the abundance. A correlation heat map was created to visualize the correlation among infiltrating immune cells. Furthermore, the correlation of critical genes with the differential immunity cells was analyzed.

### 2.6 WGCNA in GSE57148

Weighted gene co-expression network analysis (WGCNA) was performed to construct modules with similar expression pattern genes on GSE57148 (15). Firstly, outlier samples and low-expressed genes were eliminated. Secondly, the adjacency matrix was built based on an optimal soft threshold and converted into a topological overlap matrix. Then, a hierarchical clustering tree was constructed to classify the highly co-expressed genes into the same module. Finally, COPD and neutrophils were identified as characteristics to compute the correlation between gene modules and traits.

### 2.7 DEGs detection in GSE57148

COPD samples (n = 96) and normal samples (n = 90) in the GSE57148 dataset were analyzed using the limma package to screen for differentially expressed genes (DEGs), with an adjusted p-value of < 0.05 and | log2(fold change, FC) | > 0.4 as thresholds.

### 2.8 Functional enrichment

GO analysis was a popular approach for massive functional enrichment, encompassing biological process (BP), cellular component (CC), and molecular function (MF). KEGG was a globally accessible database storing essential data on genomes, biological pathways, diseases, and drugs. GO terms and KEGG pathway analysis were annotated for gene function by the clusterProfiler package, and the top-ranked results were visualized (16).

### 2.9 Mendelian randomization analysis between genes and COPD

The MR analysis was to investigate the causality between DEGs and the risk of COPD, where the instrumental variables (IVs) were single nucleotide polymorphisms (SNPs). The cis-eQTL data of DEGs were obtained from eQTLGen, and GWAS statistics for COPD outcome were obtained from integrated epidemiology unit (IEU) database (ukb-b-20464) (17, 18). The cis-eQTL data were used as exposure factors and SNPs with P-value < 5 × 10^–8^ were used for the MR analysis. If no suitable SNPs were available at the stringent threshold, the threshold was loosened to 5 × 10^–6^. There were still no relevant SNPs, so this cis-eQTL data was not used for MR analysis. All SNPs were identified 100 kb upstream and downstream of the corresponding gene location, with a threshold for Linkage disequilibrium (LD) of R2 < 0.2 and a physical distance threshold of 250 kb.

The TwoSampleMR package performed MR analysis. MR estimates were calculated for each SNP using the Wald ratio method. If more than one SNP was available, MR estimates were weighted by the inverse variance of the ratio estimates (inverse variance weighted, IVW). Cochran’s Q statistic was used to test for heterogeneity (19). When the number of SNPs was no less than three, MR-Egger regression evaluated potential horizontal pleiotropy (20).

### 2.10 PPI analysis

The protein-protein interaction (PPI) network was built on the STRING online site (21). An interaction score greater than 0.4 (medium confidence score) was considered meaningful. Then, the PPI network was further analyzed in Cytoscape. CytoHubba, a Cytoscape plugin that discovered hub terms and sub-networks from interaction groups, was used to analyze PPI network hub genes. The top 10 genes were confirmed using the maximal clique centrality (MCC) computing method with the Cytohubba plugin.

### 2.11 Quantitative real-time polymerase chain reaction in leukocytes

Peripheral blood samples in this study were obtained from Tongji Hospital, Wuhan, China. All subjects signed an informed consent form. The Ethics Committee of Tongji Hospital, Huazhong University of Science and Technology had approved this research.

Peripheral blood was centrifuged to obtain blood cells, which were treated with erythrocyte lysis buffer (TBD, China) to obtain leukocytes for the assay. The total RNA of leukocytes was extracted with RNAiso plus kit (Takara, Japan), followed by reverse transcription to cDNA using cDNA RT-PCR kit (Takara, Japan). qRT-PCR was performed using a CFX Connect Real-Time System (Bio-Rad, USA) with SYBR Premix Ex Taq (Takara, Japan) and the specific primers. The primers (Sangon Biotech, China) were shown in **Table 1**:

**Table 1:** Primer sequences for validating genes.

Gene name	Primer sequence (5’-3’)	
	Forward	Reverse
ACTB	AGAAAATCTGGCACCACACCT	AGAAAATCTGGCACCACACCT
IL1B	GCCCTAAACAGATGAAGTGCTC	GAGATTCGTAGCTGGATGCC
MCL1	TAACAAACTGGGGCAGGATT	TCCCGTTTTGTCCTTACGAG
PTGS2	CTGGCGCTCAGCCATACAG	CGCACTTATACTGGTCAAATCCC
CXCL2	GGCAGAAAGCTTGTCTCAACCC	CTCCTTCAGGAACAGCCACCAA
NAMPT	ATCCTGTTCCAGGCTATTCTGT	CCCCATATTTTCTCACACGCAT
CDKN1A	CCCCATATTTTCTCACACGCAT	GGTTCTGACGGACATCCCCA
EGR1	AGCAGCACCTTCAACCCTCAGG	GAGTGGTTTGGCTGGGGTAACT
SOD2	CTGGACAAACCTCAGCCCTAAC	AACCTGAGCCTTGGACACCAAC
CXCL3	TTCACCTCAAGAACATCCAAAGTG	TTCTTCCCATTCTTGAGTGTGGC
TNFAIP3	CTCAACTGGTGTCGAGAAGTCC	TTCCTTGAGCGTGCTGAACAGC
ZFP36	GACTGAGCTATGTCGGACCTT	GAGTTCCGTCTTGTATTTGGGG

### 2.12 Regulatory network construction

The NetworkAnalyst online tool was used to construct a gene regulation network of the hub gene–transcription factor interaction network. The transcription factor data were obtained from the JASPAR database to build the network (22, 23). Subsequently, the results were visualized using Cytoscape.

### 2.13 Statistical analysis

All data was analyzed by R (version 4.1.3) and GraphPad Prism 8 Software. For normally and non-normally distributed data, we used an unpaired t-test and a non-parametric test to compare differences between groups, respectively. Pearson correlation analysis was used to estimate the correlation. Unless explicitly stated, the p-value less than 0.05 was considered statistically significant.

## 3 Results

### 3.1 Quality control and dimension reduction in scRNA-seq GSE173896

**Figure 1:**
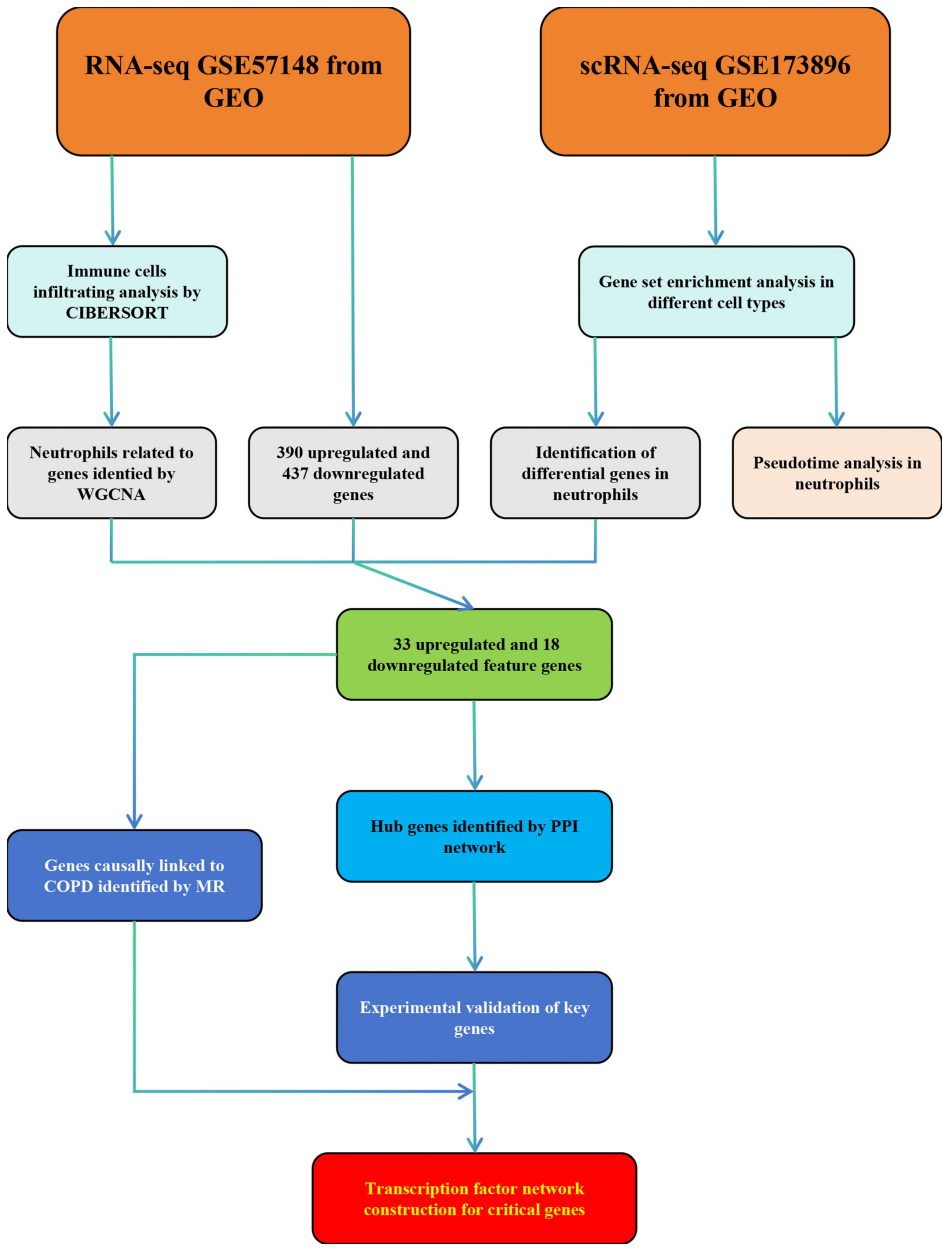
Flow chart of this study.

**Figure 2:**
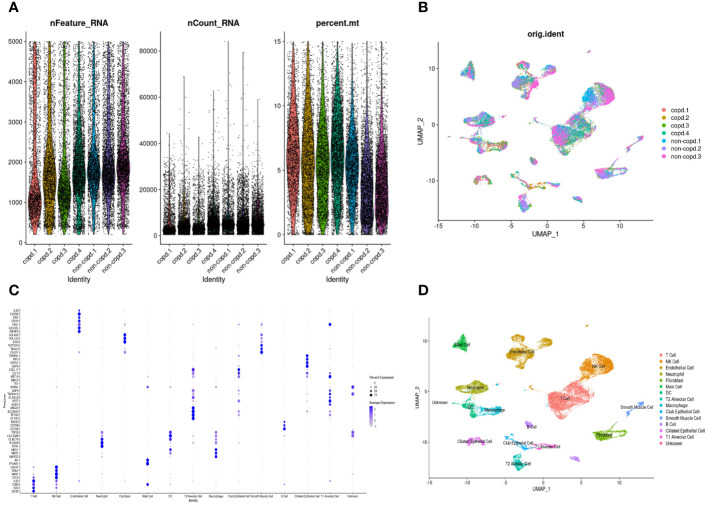
Quality control and dimension reduction in scRNA-seq GSE173896. **(A)** The proportion of mitochondrial genes was adjusted to ensure the quality of cell samples. The proportion of mitochondrial genes per cell was less than 15%. **(B)** UMAP dimensional plot after adjusting for batch effects. **(C)** Dot plot of z-scores for marker gene expression values. Dot size reflected the percentage of cells with gene expression; color corresponded to the magnitude of gene expression. **(D)** UMAP of 14 cell types in scRNA-seq GSE173896.

The workflow of this study was presented in **Figure 1**. A quality control procedure was performed on the scRNA-seq dataset. As shown in **Figure 2A**, specific low-quality cells were excluded based on their gene expression profile to avoid misinterpretation of the data. The mitochondrial proportion was used as a threshold to filter out low-quality cells. The CCA algorithm integrated the datasets from four COPD patients and three controls (**Figure 2B**). According to the expression level of marker genes (**Figure 2C**), all cells were classified as 16 clusters and annotated into 14 cell types based on the marker genes: T cells (marked with CD3D, CD2, and CD69), neutrophils (FCN1 and S100A9), dendritic cells (TGFBI and HLA-DMB), mast cells (KIT and TPSAB1), NK cells (CCL5, NKG7, GNLY, and CD247), B cells (CD79A, CD79B, and IGHG3), macrophages (MARCO, MSR1, and MRC1), alveolar type 1 cells (AGER, KRT7, and TSPAN13), alveolar type 2 cells (SFTPD, SFTA2, SLC34A2, and ABCA3), club epithelial cells (BIRC5, KRT19, and CXCL17), ciliated epithelial cells (CAPS, RSPH1, PIFO, and TSPAN1), endothelial cells (CALCRL and RAMP2), fibroblasts (COL1A2 and COL6A2), and smooth muscle cells (CALD1, TAGLN, and NOTCH3)(**Figure 2D**).

### 3.2 Single-cell rank-based gene set enrichment analysis

**Figure 3:**
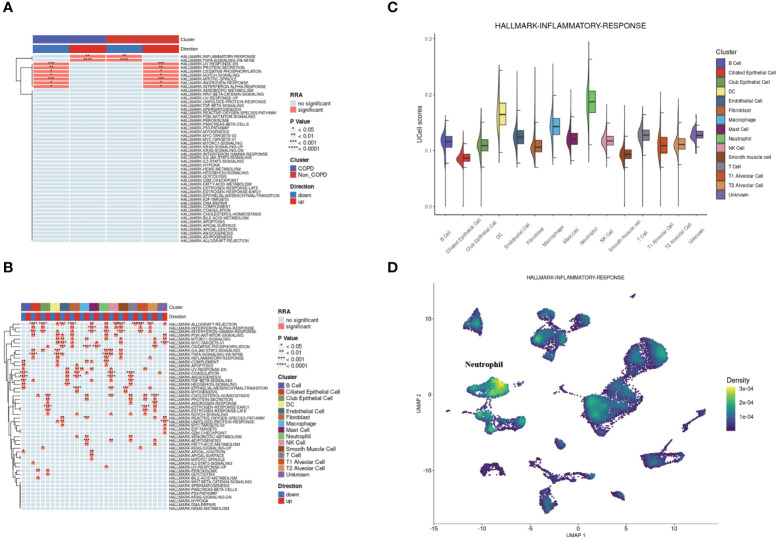
Single-cell rank-based gene set enrichment analysis. **(A)** Heatmap plot of co-upregulated or co-downregulated gene sets per group in RRA. “INFLAMMATORY-RESPONSE” and “TNFA-SIGNALING-VIA-NFKB” were upregulated in the COPD group. **(B)** Heatmap plot of co-upregulated or co-downregulated gene sets per cell type in RRA. “INFLAMMATORY-RESPONSE” and “TNFA-SIGNALING-VIA-NFKB” were upregulated in neutrophils and DCs. **(C)** Half vlnplot of “HALLMARK-INFLAMMATORY-RESPONSE” in Ucell among clusters. **(D)** Density scatterplot of “HALLMARK-INFLAMMATORY-RESPONSE” in Ucell on UMAP plot. RRA, robust rank aggregation.

According to the hallmark gene sets collected from the MsigDB database, we used the irGSEA package to calculate each cell’s gene set enrichment score. Overall, there were two hallmark gene sets upregulated in COPD samples: INFLAMMATORY-RESPONSE and TNFA-SIGNALING-VIA-NFKB. At the same time, the two gene sets were significantly downregulated for control samples (**Figure 3A**). Next, the same operation was on different types of cells. The two gene sets were both upregulated in neutrophils and DCs (**Figure 3B**). INFLAMMATORY_RESPONSE gene set was significantly enriched in neutrophils (**Figure 3C** and **Figure 3D**).

### 3.3 Enrichment analysis and pseudotime analysis of neutrophils in the single-cell dataset GSE173896

**Figure 4:**
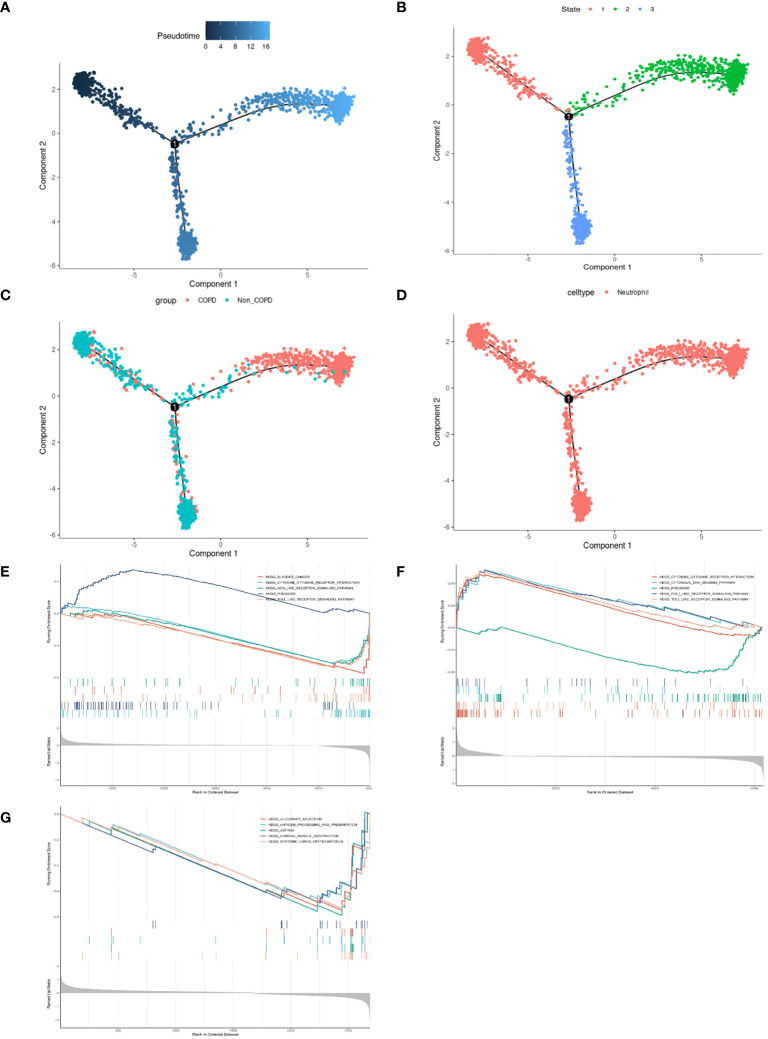
Pseudotime analysis and enrichment analysis of neutrophils in scRNA-seq GSE173896. **(A)** Timing differences in cell differentiation. Darker blue represented an earlier stage of differentiation, while lighter blue indicated a later stage of differentiation. This served as a starting point for subsequent analysis. **(B)** Three stages of neutrophil differentiation. State 1 was the earliest stage of differentiation, and state 2 was the latest. **(C)** Differences in differentiation between COPD neutrophils and normal neutrophils. The turquoise represented control, and the red represented COPD. **(D)** All cells analyzed were neutrophils. **(E)** GSEA of DEGs on KEGG in state.1. **(F)** GSEA of DEGs on KEGG in state.2. **(G)** GSEA of DEGs on KEGG in state.3.

Neutrophilic inflammation predominates in the pathogenesis of COPD. The FindMarkers method was employed to identify genes differentially expressed in neutrophils from COPD patients and controls, revealing many inflammatory and immune responses-associated biological processes in COPD (**Supplementary Figure 1**). Thus, we performed a simulation analysis on the cell trajectory differentiation of all neutrophils. It was shown that the darker the blue color, the earlier the cells differentiated, indicating that the neutrophils differentiate from the left to the right with the differentiation of time, and the latest differentiated cell was represented by the lightest blue (**Figure 4A**). Neutrophils labeled with different colors had three differentiated states, with the red one on the left being the earliest differentiated type (**Figure 4B**). The differentiation process between COPD and control neutrophils was then investigated, and the results showed that COPD neutrophils differentiated later, whereas control neutrophils differentiated earlier (**Figure 4C**). All the cells analyzed were neutrophils (**Figure 4D**). The GSEA was conducted in neutrophils of different differentiation states to investigate the relationships between pseudotime and gene functions and showed that the latest differentiated neutrophils were mainly associated with the upregulation of inflammatory and immune responses (**Figures 4E–G**, **Supplementary Figure 2**).

### 3.4 Immune cells infiltrating analysis in GSE57148

**Figure 5:**
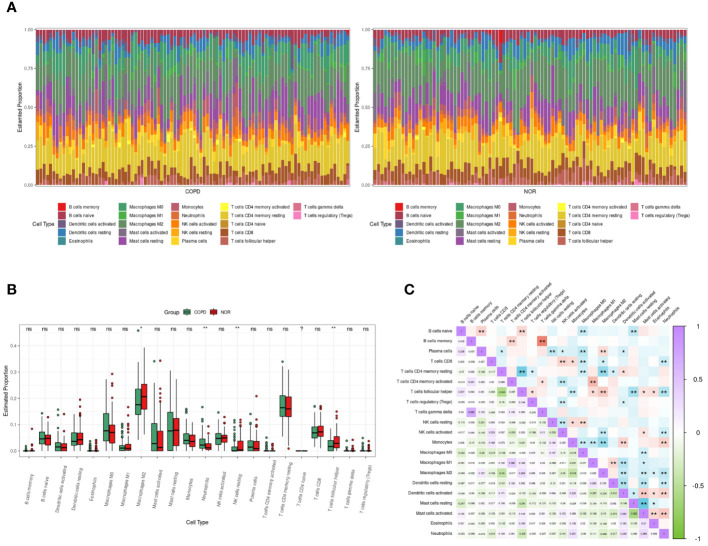
Immune cells infiltrating analysis. **(A)** The enrichment fraction of 22 types of immune infiltrating cells in the COPD and normal samples. **(B)** Box plot of 22 types of immune infiltrating cells in the COPD and normal samples. Normal samples were denoted by red, whereas COPD samples were denoted by green. **(C)** The heat map of the correlation among immune infiltrating cells. The color shade of the squares indicated the strength of the connection; purple indicated a positive correlation, while green indicated a negative correlation. The darker the color, the stronger the correlation; *P < 0.05, **P < 0.01.

The RNA sequencing dataset GSE57148 included 98 COPD and 91 normal samples. The cell type proportions in each sample were calculated by the CIBERSORT algorithm, and different colors represented different immune cells (**Figure 5A**). There were differences in the proportions of four types of immune cells, including neutrophils, M2-type macrophages, resting NK cells, and T follicular helper cells, in COPD samples and normal samples. Furthermore, the proportion of neutrophils was significantly increased in COPD samples (**Figure 5B**). In **Figure 5C**, the correlation heatmap indicated that neutrophils correlated negatively with Dendritic cells resting (p<0.01, r= -0.217), CD8 T cells (p<0.01, r= -0.216), T follicular helper cells (p<0.01, r= -0.305), NK cells activated (p<0.01, r= -0.191), and M2 type macrophages (p<0.01, r= -0.246). Conversely, a positive correlation between neutrophils and three cells was demonstrated, including Dendritic cells active (p<0.01, r= 0.236), monocytes (p< 0.01, r= 0.251), and Mast cells activated (p<0.01, r= 0.289).

### 3.5 Weighted gene co-expression network analysis in GSE57148

**Figure 6:**
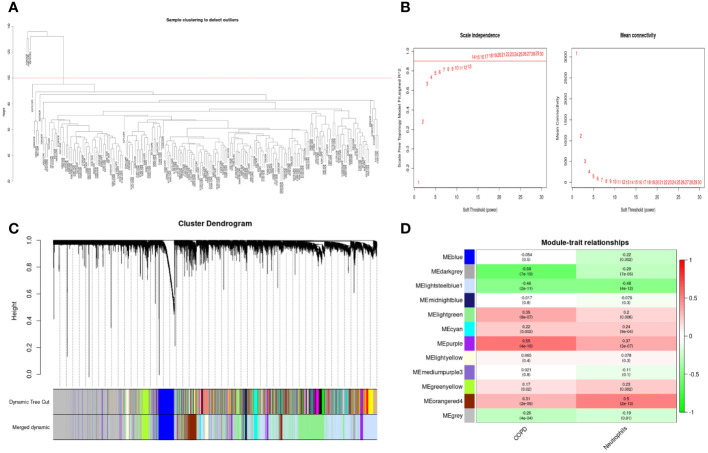
Weighted gene co-expression network analysis in GSE57148 dataset. **(A)** Sample clustering of GSE57148 to detect outliers. Three outliers were excluded, and all remaining clusters were selected for further analysis. **(B)** The optimal soft-threshold power. The threshold was 14. **(C)** Clustering dendrograms of genes based on a dissimilarity measure (1-TOM). Different modules were produced and shown in different colors by aggregating genes with strong correlations into the same module. **(D)** Correlations between module eigengenes and sample traits evaluated module-trait associations. The lightssteelblue1 module showed a significant negative correlation with neutrophils (COR = -0.48, P < 0.001) and a significant negative correlation with COPD (COR = -0.46, P < 0.001). The orangered4 module was significantly positively correlated with neutrophils (COR = 0.5, P < 0.001) and positively correlated with COPD (COR = 0.31, P < 0.001). Genes in the lightssteelblue1 and orangered4 modules were labeled as WGCNA-hub genes.

As shown in **Figure 6A**, the clustering tree for samples screened out three abnormal samples, and 186 samples (96 COPD and 90 normal samples) remained for further analysis. Sequentially, based on the scale-free network, the best soft-threshold power of 14 was chosen. (**Figure 6B**). These genes were clustered into 12 modules based on their similar co-expression characteristics (**Figure 6C**). The correlation analysis of modules and traits (neutrophils and COPD) illustrated that the orangered4 module and lightssteelblue1 module were significantly associated with neutrophils (p<0.001, r= 0.5; p<0.001, r= -0.48) and COPD (p<0.001, r= 0.31; p<0.001, r= -0.46) (**Figure 6D**). Therefore, the genes in the orangered4 module and lightssteelblue1 module were selected as essential genes relevant to neutrophils for further analysis.

### 3.6 Identification of DEGs

**Figure 7:**
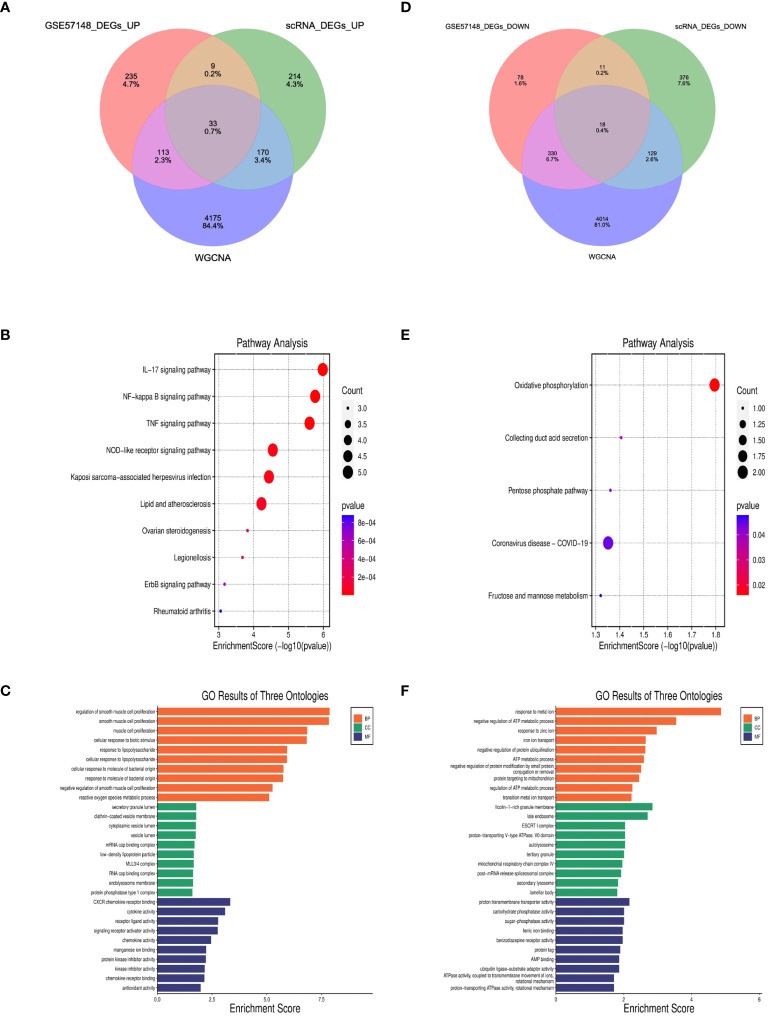
Identification of DEGs and enrichment analysis of DEGs. **(A, D)** The intersection of single cell DEGs, WGCNA, and bulk RNA sequencing DEGs in Venn diagram. **(B, C)** GO and KEGG results of upregulated DEGs. The x-axis indicated the enrichment score associated with the terms, while the y-axis indicated the pathway terms. **(E, F)** GO and KEGG analyses of downregulated DEGs.

827 DEGs were identified between COPD and normal samples in GSE57148, including 390 upregulated and 437 downregulated genes (**Supplementary Figure 3**). Venn diagrams were performed to obtain the DEG profiles intersection of scRNA-seq DEGs, RNA-seq, and WGCNA model genes, and 33 upregulated and 18 downregulated feature genes in total were remarkably expressed differentially in all 3 groups (**Figures 7A, D**, **Supplementary Table 1**). KEGG pathway analysis revealed that the upregulated DEGs were mainly enriched in the IL-17 signaling pathway, NF-kappa B signaling pathway, and TNF signaling pathway. Meanwhile, the downregulated DEGs were primarily enriched in oxidative and collecting duct acid secretion (**Figures 7B, E**). The GO analysis showed that the upregulated DEGs were enriched in smooth muscle proliferation, secretory, and cytokine activity (**Figure 7C**). The downregulated DEGs were enriched in response to the metal ion, late endosome, and proton transmembrane transporter activity. (**Figure 7F**).

### 3.7 MR analysis between feature DEGs and COPD

The MR analysis was used to explore the relationship between feature DEGs and COPD to verify whether the relationship was causal or correlative. Among these 51 feature DEGs, 9 genes were eliminated without suitable SNPs, and 2 genes had SNPs with P-value < 5 × 10^–6^. **Table 2** and **Supplementary Table 2** displayed the causal effect of each feature DEG on COPD. IVW analysis reveals that NAMPT (OR=1.0102, p=0.0064), PTGS2(OR=1.0139, p=0.0161), SLC2A3(OR=1.0052, p=0.0014) and TRIB1(OR=1.0172, p=0.0405) levels were associated with an increased risk of COPD, while CDKN1A (OR=0.9962, p=0.0169), and CSRNP1(OR=0.9933, p=0.0384) were related to a decreased risk of COPD. Information on SNPs and other MR estimates for 6 genes was shown in **Supplementary Table 3**. The Cochran’s Q test revealed no heterogeneity in these gene results. MR-Egger regression analysis indicated no horizontal pleiotropy in the results of these genes (**Table 2**).

**Table 2:** MR analysis between feature DEGs and COPD.

Gene	Nsnp	Method	P.value	OR (95% CI)	Heterogeneity_P.val	Pleiotropy_P.val
CDKN1A	10	Inverse variance weighted	0.0169	0.9962(0.9931 to 0.9993)	0.6403	0.9498
CSRNP1	5	Inverse variance weighted	0.0384	0.9933(0.987 to 0.9996)	0.7111	0.9379
NAMPT	5	Inverse variance weighted	0.0064	1.0102(1.0028 to 1.0175)	0.8456	0.4242
PTGS2	1	Wald ratio	0.0161	1.0139(1.0026 to 1.0254)		
SLC2A3	8	Inverse variance weighted	0.0014	1.0052(1.002 to 1.0083)	0.8848	0.5695
TRIB1	1	Wald ratio	0.0405	1.0172(1.0007 to 1.0338)		

P.value<0.05 was considered statistically significant

OR, odds ratio; CI, confidence interval.

### 3.8 PPI network analysis and identification of hub genes

**Figure 8:**
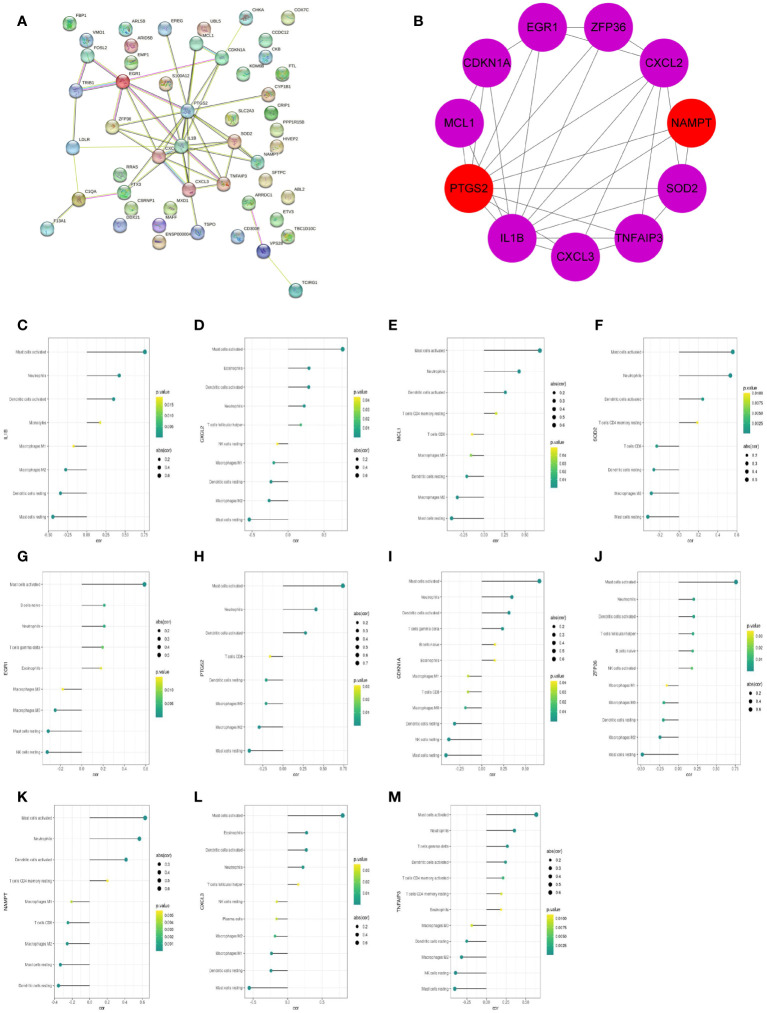
The PPI networks and hub genes were analyzed using the STRING database and Cytoscape software. **(A)** PPI network display. **(B)** Top 10 hub genes explored by CytoHubba. **(C–M)** The correlation of 11 hub genes (IL1B, CXCL2, MCL1, SOD2, EGR1, PTGS2, CDKN1A, ZFP36, NAMPT, CXCL3, TNFAIP3) with the immune cells. The dot size represented the degree of correlation between the gene and the immune cell. The dot color indicated the P-value; the greener the color, the smaller the P-value.

The protein–protein interaction (PPI) network of 51 feature DEGs was constructed based on the STRING online database (**Figure 8A**). The interaction networks were visualized in Cytoscape and contained 25 nodes and 47 edges. Furthermore, we implemented the cytoHubba plugin to search for the most significant hub genes by the maximal clique centrality algorithm (**Figure 8B**). The top 10 hub genes were identified, including IL1B, CXCL2, PTGS2, MCL1, SOD2, EGR1, CDKN1A, CXCL3, NAMPT, TNFAIP3, and ZFP36, and both NAMPT and MCL1 ranked 10th. The 11 hub genes were all upregulated genes. Correlation analysis revealed that these hub genes had a correlation with various immune cells, and all of them were significantly positively correlated with neutrophils (all P<0.05) (**Figures 8C–M**).

### 3.9 Validations of the hub genes

**Figure 9:**
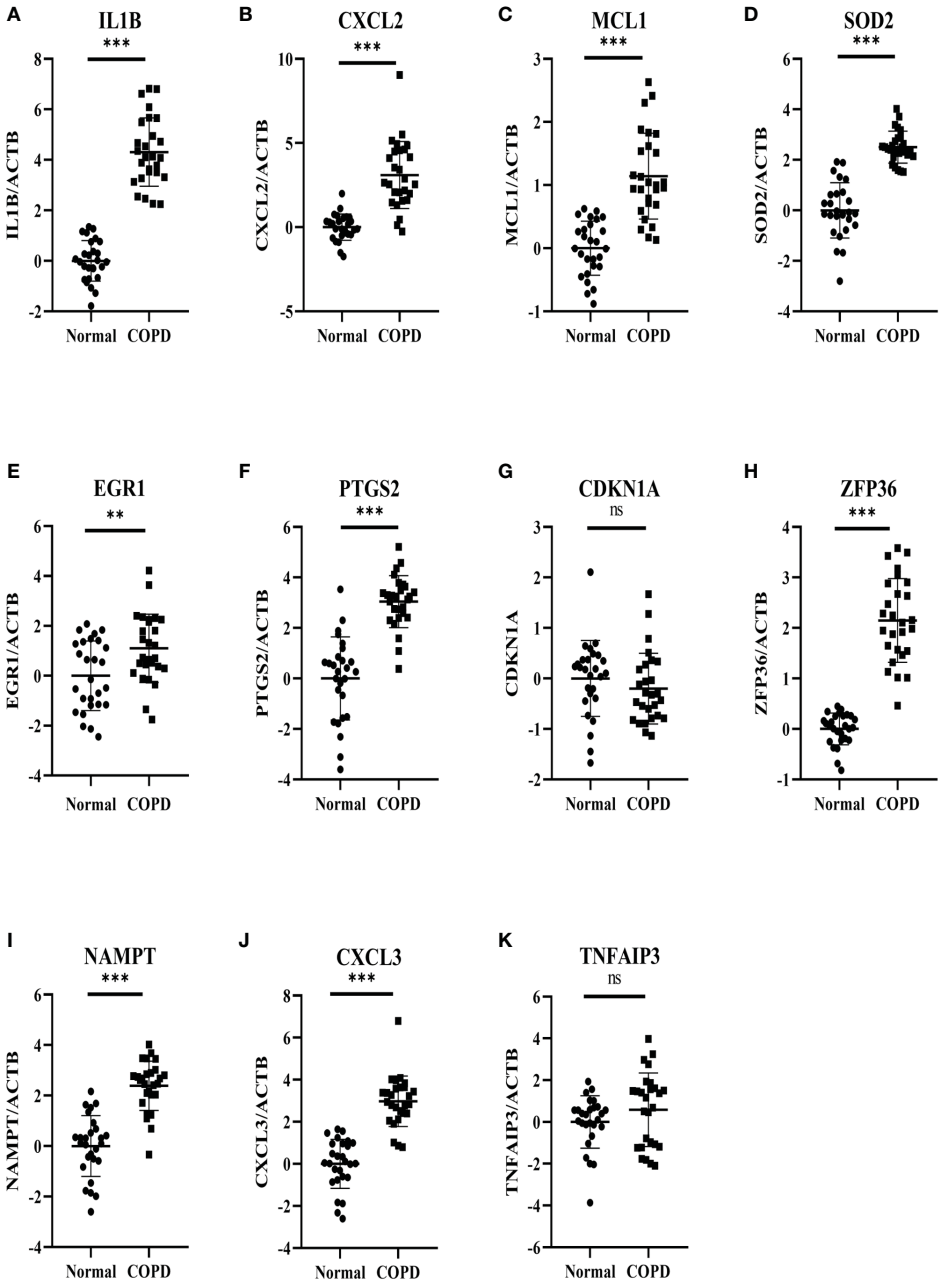
Experimental verification of the hub genes. The relative mRNA expressions of 11 genes in the COPD subjects and normal subjects were shown, including **(A)** IL1B, **(B)** CXCL2, **(C)** MCL1 **(D)** SOD2, **(E)** EGR1, **(F)** PTGS2, **(G)** CDKN1A, **(H)** ZFP36 **(I)** NAMPT, **(J)** CXCL3, **(K)** TNFAIP3. Results were expressed as mean ± SD; n=27 for normal and n=27 for COPD; **P < 0.01, ***P < 0.001 ns, no significance.

**Figure 10:**
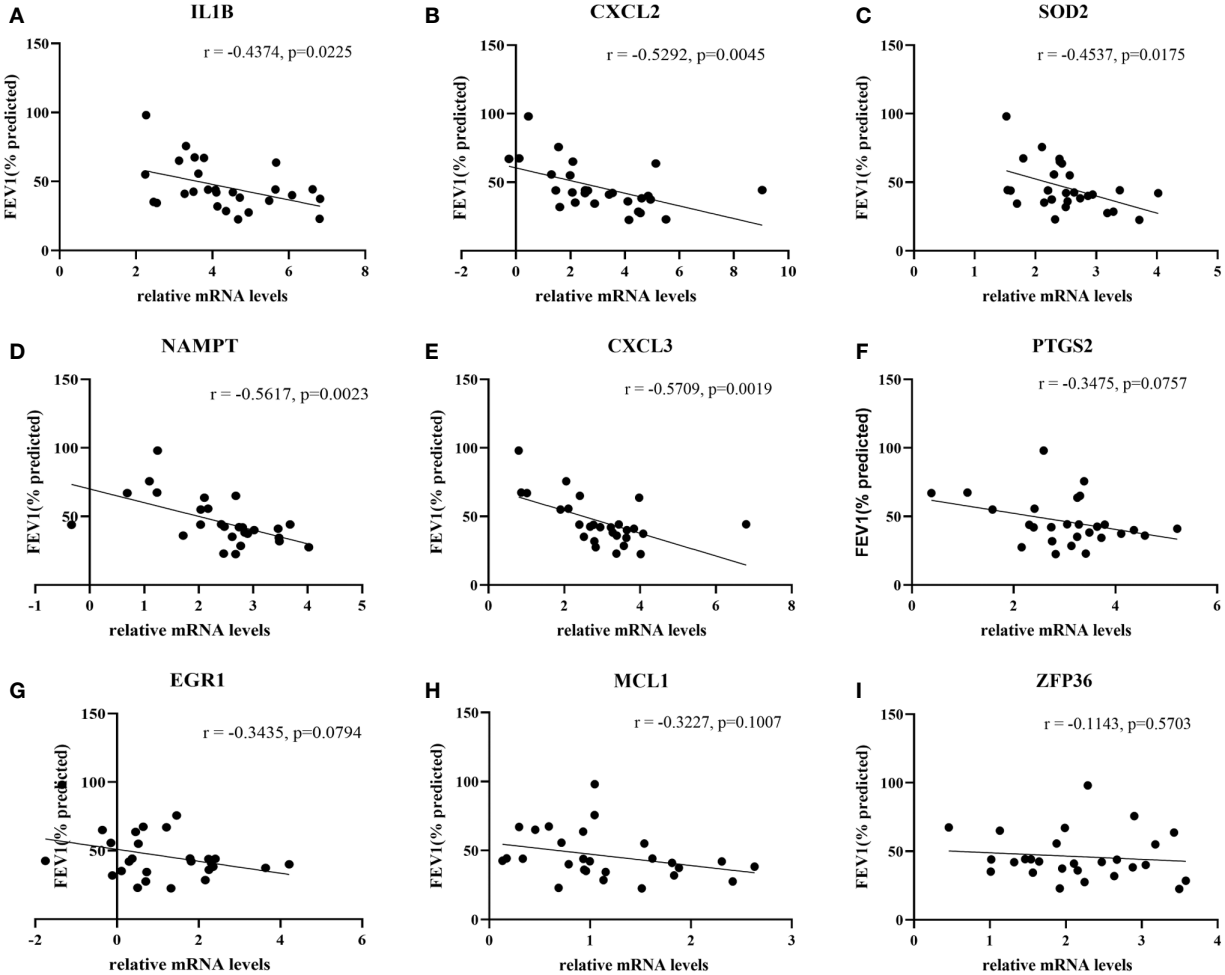
The correlation analysis between hub genes and lung function in COPD patients. The lung function was negatively associated with **(A)** IL1B expression and **(B)** CXCL2 expression, **(C)** SOD2 expression and **(D)** NAMPT expression, **(E)** CXCL3 expression, whereas no significant associations with the expressions of **(F)** PTGS2, **(G)** EGR1, **(H)** MCL1 and **(I)** ZFP36.

27 COPD patients and 27 healthy individuals provided peripheral blood samples. Since neutrophils make up about 70% of all leukocytes in peripheral blood, peripheral blood leukocytes were isolated from blood samples. The qRT-PCR in leukocytes was performed to verify hub genes. In **Table 3**, the clinical characteristics of two group subjects were compared. Compared with normal subjects, the mRNA levels of IL1B, CXCL2, PTGS2, MCL1, SOD2, EGR1, NAMPT, CXCL3, and ZFP36 were markedly higher in COPD patients (**Figure 9**). Moreover, correlation analysis revealed that IL1B, CXCL2, SOD2, NAMPT, and CXCL3 were inversely correlated with lung function in COPD patients (r = -0.4374, p<0.05; r = -0.5292, p<0.01; r = -0.4537, p<0.05; r = -0.5617, p<0.01; r = -0.5709, p<0.01; respectively) (**Figure 10**).

**Table 3:** Overall subjects characteristics.

	Normal	COPD	P-value
Subjects	27	27	
Age, y	61.70 (± 5.876)	64.26 (± 8.596)	0.2079
Male, %	27 (100%)	27 (100%)	
BMI	23.23 (± 3.916)	21.74 (± 3.698)	0.0952
FEV_1_/FVC		41.13 (± 11.13)	
FEV_1_% pred		46.18 (± 17.38)	

Data are shown as percentages and means ± SD.

BMI, Body mass index; FEV_1_, Forced expiratory volume in the first second.

FVC, Forced vital capacity.

### 3.10 The regulatory network construction of NAMPT and PTGS2

**Figure 11:**
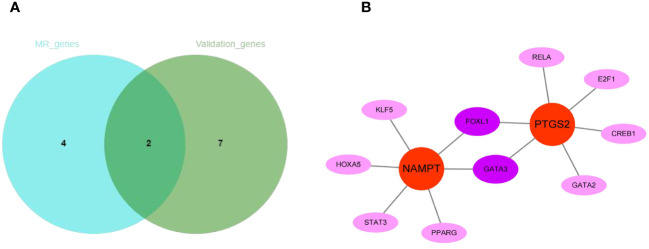
The regulatory network construction of NAMPT and PTGS2. **(A)** The intersection of MR-genes and validation-genes. Two hub genes (NAMPT and PTGS2) were identified. **(B)** Interaction network of TFs and genes for the two hub genes.

The intersection of 6 genes identified by Mendelian randomization and 9 genes elevated in peripheral blood leukocytes of COPD patients identified 2 more crucial genes, NAMPT and PTGS2 (**Figure 11A**). Furthermore, NAMPT was negatively correlated with lung function in COPD patients. Using the JASPAR database, a network of transcription factors (TFs) for two genes was constructed. TF network showed that 10 TFs were obtained, of which 2 were GATA3 and FOXL1 with degree ≥ 2. (**Figure 11B**).

## 4 Discussion

COPD remains a significant global health challenge. Despite the best current therapies, patients with COPD have an alarmingly high mortality rate and often have limited therapeutic options. In 2019, the number of deaths and DALYs due to COPD globally was 3.28 million and 74.43 million, respectively (4). Therefore, it is critical to identify key molecular targets of cellular mechanisms in COPD. As demonstrated by numerous RCT trials (e.g., pivotal clinical trials of roflumilast and dupilumab, etc.), identifying these targets can greatly elucidate therapeutic regimens for individual patients, thereby facilitating the development of precision medicine (24). Neutrophil inflammation plays a central role in the pathophysiology of COPD and persists in the entire disease process, but targets for its action remain relatively scarce, making the identification of new biomarkers of neutrophilic inflammation critical to developing effective therapeutic options for COPD.

Neutrophils and their products are considered to be significant contributors to the inflammatory changes in the airways of COPD patients (25). During the development of COPD, neutrophils become overstimulated, and their activation and inhibition pathways are altered at the molecular level. This leads to increased infiltration, prolonged lifespan, heightened activation, and increased necrosis. These changes result in elevated levels of neutrophil products in the lungs, which can cause lung injury (26). Furthermore, the quantity and product of neutrophils in the blood and lung tissue correlate with the severity of the disease, as reflected in lung function (e.g., degree of airway obstruction and decline in FEV1) (26).

In this study, the scRNA-seq analysis of lung tissues from COPD patients and normal controls revealed that the genes in the inflammatory response pathway were significantly upregulated and enriched in neutrophils in COPD. In trajectory and enrichment analysis, COPD neutrophils differentiated later than from normal controls, implying a series of changes in the neutrophil state during the development of COPD. The latest differentiated neutrophils were mainly associated with the upregulation of inflammatory and immune responses, suggesting the reciprocal relations among neutrophils, inflammation, and COPD. It had been found that in the development of COPD, neutrophils became over-infiltrated, which led to the accumulation of large amounts of inflammatory modulators, including proteases associated with tissue damage and chemoattractants that recruit additional inflammatory cells (27). At the same time, neutrophils in COPD responded differently to many of the chemical signals regulating inflammation and immune responses compared to healthy individuals (26). To identify the underlying hub genes in the neutrophilic inflammation of COPD, immune infiltration analysis, WGCNA, and differential expression analysis were performed, and 51 neutrophil-related DEGs were selected. Next, 6 genes causally associated with COPD were identified by Mendelian randomization analysis, of which 4 genes were risk factors for COPD and 2 genes were protective factors. Meanwhile, 11 hub genes were identified by PPI analysis, 9 of which were markedly upregulated in COPD patients. Moreover, 5 genes were inversely correlated with lung function in patients with COPD. Finally, establishing a regulatory network of transcription factors related to NAMPT and PTGS2 could help explore important mechanisms of neutrophilic inflammation in COPD.

MCL-1 and EGR1 can further exacerbate airway inflammation in COPD. MCL-1 is an anti-apoptotic protein of the BCL-2 family (28).It is believed to prevent neutrophil apoptosis by binding to pro-apoptotic BCL-2 proteins, resulting in prolonged inflammation in the lung and airway (29).EGR-1 is an instant early responsive gene encoding a zinc finger transcription factor upregulated in mature neutrophils (30). It has been found to be consistently upregulated in advanced emphysema, exacerbating cigarette smoke-induced lung inflammation (31).IL1B and CXCL2 may also have an association with lung function in COPD. IL-1B, a member of the interleukin-1 family involved in neutrophil recruitment, may serve as a biomarker of persistent neutrophilic airway inflammation and potentially persistent exacerbations of COPD (32). It also has a significant negative correlation with lung function, such as predicted FEV1 percent and FEV1/FVC ratio (33). CXCL2 is a member of the chemokine CXC family, which can bind to CXCR2 on the surface of neutrophils to promote neutrophil migration (34). Up-regulation of CXCL2 expression promotes lung inflammation and increases airflow obstruction in COPD (35, 36). SOD2, also known as manganese superoxide dismutase, is a mitochondrial antioxidant enzyme that plays a bidirectional role in COPD (37). One study found that sputum SOD activity is a marker of COPD exacerbation, while another study concluded that SOD2 ameliorates mitochondrial oxidative stress and cellular damage in COPD (38, 39).ZFP36 encodes triple trehalose (TTP), which transcriptionally inhibits many pro-inflammatory factors associated with COPD and may regulate neutrophil homeostasis (40).The up-regulation of ZFP36 expression was associated with a reduction in lung inflammation, airway remodeling, and emphysema-like alveolar enlargement, which may suggest a protective role for ZFP36 in the development of COPD (41). Similar to CXCL2, CXCL3 is also an inflammatory factor of the CXC chemokine family and can bind to CXCR2 on the surface of neutrophils (42, 43). A previous scRNA-seq study on lung tissue in septic lung injury revealed that the Neutrophil-CXCL3-High subpopulation was closely related to the hyperinflammatory response (44). So far, the function of CXCL3 in COPD is poorly understood. This study may offer a new insight into the role of CXCL3 in the neutrophilic inflammation of COPD.

Among the 9 hub genes, NAMPT and PTGS2 were further confirmed by MR analysis as risk factors for COPD. NAMPT is a specific enzyme with cytokine-like characteristics that have been shown to play a substantial role in inflammation, cellular metabolism, and immune regulation (45, 46). Intracellularly, it catalyzes the conversion of nicotinamide into NAD. However, extracellularly, it acts as a cytokine to convey various signals, including promoting myeloid cell differentiation/polarization, activating inflammatory vesicles, and secretion of pro- or anti-inflammatory cytokines (47). During G-CSF-stimulated granulopoiesis, NAMPT was essential for regulating neutrophil development and functionality (48, 49). In tumors, breast tumor derived NAMP activated SIRT1 in neutrophils, which polarized neutrophils into tumor-associated aged neutrophils, thus promoting tumor metastasis (50). In addition, NAMPT functioned as a novel inflammatory cytokine in LPS-stimulated sepsis and exerted inhibitory effects on the apoptosis of neutrophils by decreasing the activity of caspase-3 and caspase-8 (51). NAMPT has also been reported to regulate CXCL2 to recruit neutrophils to exacerbate neutrophil-induced tissue injury in myocardial infarction (52). Meanwhile, intracellular NAMPT exerts its function in LPS-induced neutrophil extracellular traps-related diseases (53). In this study, NAMPT was significantly upregulated in COPD neutrophils and may be involved in neutrophil inflammation in the development of COPD through the related mechanisms, which may be helpful in the diagnosis and treatment of COPD.

PTGS2, also known as cycloxygenase-2 (COX-2), is a key enzyme in promoting the rate-limiting step of prostaglandin synthesis (54). PTGS2 and its derivative prostaglandin E2 (PGE2) have been found to play an essential pro-inflammatory role in various diseases (55). In COPD, PGE2, the derivative of PTGS2, promotes the aggregation of neutrophils in the lungs, which can further increase the ability of neutrophils to adhere to human airway epithelial cells, thus exerting a pro-inflammatory effect (56). Meanwhile, activated neutrophils in COPD patients produce large amounts of reactive oxygen species (ROS), leading to the oxidation of arachidonic acid, which can be converted to prostaglandins through the action of COX2 and participate in the inflammatory response in COPD (57). In obese women, vascular inflammation is strongly associated with neutrophil infiltration, and COX-2 staining is highly correlated with the neutrophil marker CD66b staining, suggesting that COX2 may be associated with neutrophil inflammation (58). During acute inflammation, when neutrophils are exposed to bacterial endotoxins, cytokines, and hormones, they evoke activation of mitogen-activated protein kinase (MAPK) and nuclear factor-κB (NF-κB), which in turn promotes the expression of COX-2 and production of PGE2 (59), which also has a vital role in the regulation of inflammatory nociceptive hypersensitivity (60). Meanwhile, it has been found that bioactive implants can recruit neutrophils and trigger neutrophil extracellular traps during osseointegration, which are effective in promoting PGE2 production (61). All these facts suggest that PTGS2 is closely related to neutrophil inflammation, and the mechanism of PTGS2-induced neutrophil inflammation in COPD needs further exploration.

Although this study reveals significant findings, there are limitations. First, the number of cases we studied was relatively insufficient. This needs to be validated in a larger clinical sample. Secondly, we only studied the mRNA expression levels of the hub genes in peripheral blood leukocytes, and further validation is needed *in vivo* and *in vitro*, including the protein levels of the hub genes and their mechanisms in neutrophils in COPD development. Nevertheless, our study may provide new marker genes for neutrophilic inflammation in COPD. It may also provide a theoretical basis for further research.

## 5 Conclusion

In this research, 9 hub genes associated with neutrophil inflammation were identified by analyzing scRNA-seq and RNA-seq data in COPD and control lung tissue samples, of which 5 genes were negatively correlated with lung function in COPD patients. NAMPT and PTGS2 were further confirmed as risk factors for COPD by MR analysis, providing a novel way to detect neutrophilic inflammation and lung function in COPD. Therefore, the present study offers an idea for further research into the mechanisms of neutrophilic inflammation in COPD, which could lead to the development of new therapeutic targets.

## Data availability statement

The original contributions presented in the study are included in the article/**Supplementary Material**. Further inquiries can be directed to the corresponding author/s.

## Ethics statement

The studies involving humans were approved by Tongji Medical Ethics Committee, Tongji Medical College, Huazhong University of Science and Technology, China. The studies were conducted in accordance with the local legislation and institutional requirements. The participants provided their written informed consent to participate in this study.

## Author contributions

YHu: Writing – original draft, Writing – review & editing. YN: Writing – review & editing, Formal analysis, Software. XW: Writing – review & editing, Data curation, Resources. XCL: Writing – review & editing. YHe: Writing – review & editing. XSL: Writing – review & editing, Funding acquisition, Conceptualization, Project administration.

## Supplementary material

Supplementary Figure 1
**(A)** KEGG results of upregulated DEGs in neutrophils. The size of the bubble represented the number of enriched genes. The enriched pathways were connected to their corresponding genes with the same colored lines. Each gene’s fold change was colored according to the legend. **(B)** GO results of upregulated DEGs in neutrophils. The x-axis indicated the enrichment score associated with the terms, while the y-axis indicated the pathway terms. **(C)** KEGG results of downregulated DEGs in neutrophils. **(D)** GO results of downregulated DEGs in neutrophils.

Supplementary Figure 2 Enrichment analysis of DEGs of three stages of neutrophil differentiation in scRNA-seq GSE173896. **(A)** GSEA of DEGs on GO in state.1. **(B)** GSEA of DEGs on GO in state.2. **(C)** GSEA of DEGs on GO in state.3.

Supplementary Figure 3 Volcano plot showing DEGs in GSE57148. Blue represented low-expressed genes in COPD lung tissues, red represented high-expressed genes in COPD lung tissues, and black represented genes with no significant difference.
