# Supplementary figures and images for: Identification of novel biomarkers related to neutrophilic inflammation in COPD
Source: Front Immunol. 2024 May 30;15:1410158. doi: 10.3389/fimmu.2024.1410158 (PMC11169582; doi:10.3389/fimmu.2024.1410158)

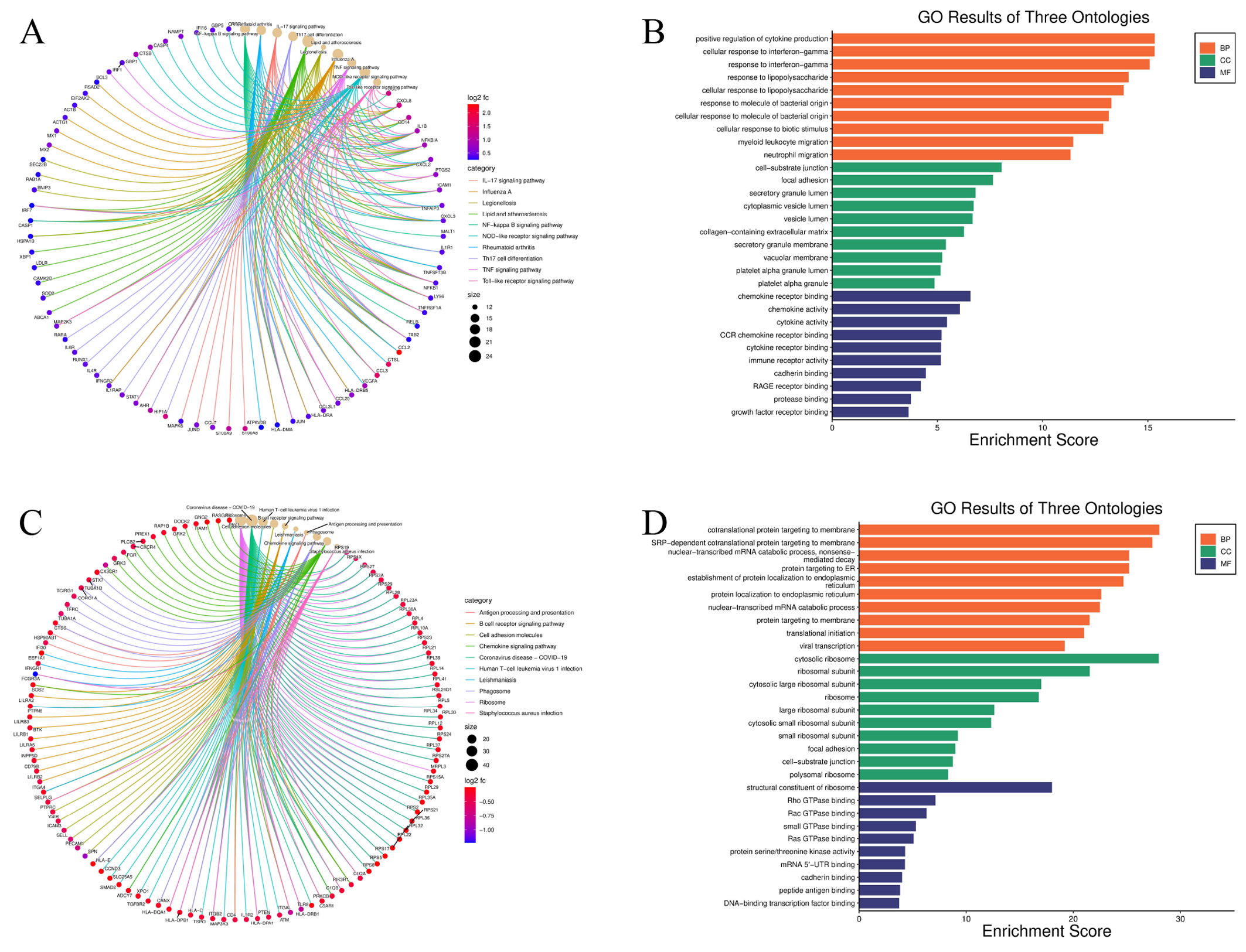

Supplement: Supplementary Figure 1 — (A) KEGG results of upregulated DEGs in neutrophils. The size of the bubble represented the number of enriched genes. The enriched pathways were connected to their corresponding genes with the same colored lines. Each gene’s fold change was colored according to the legend. (B) GO results of upregulated DEGs in neutrophils. The x-axis indicated the enrichment score associated with the terms, while the y-axis indicated the pathway terms. (C) KEGG results of downregulated DEGs in neutrophils. (D) GO results of downregulated DEGs in neutrophils. [file Image_1.tif]

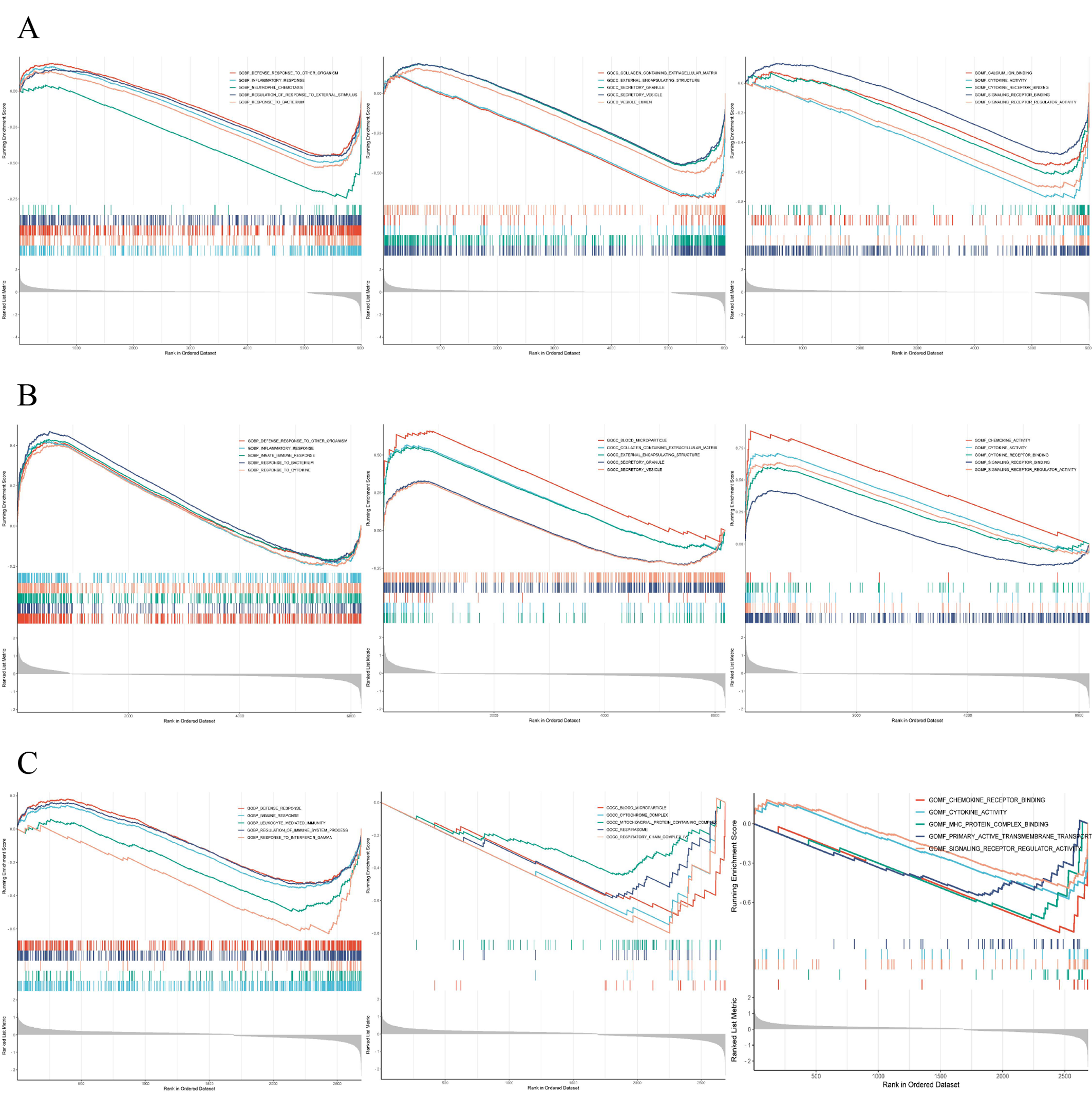

Supplement: Supplementary Figure 2 — Enrichment analysis of DEGs of three stages of neutrophil differentiation in scRNA-seq GSE173896. (A) GSEA of DEGs on GO in state.1. (B) GSEA of DEGs on GO in state.2. (C) GSEA of DEGs on GO in state.3. [file Image_2.tif]

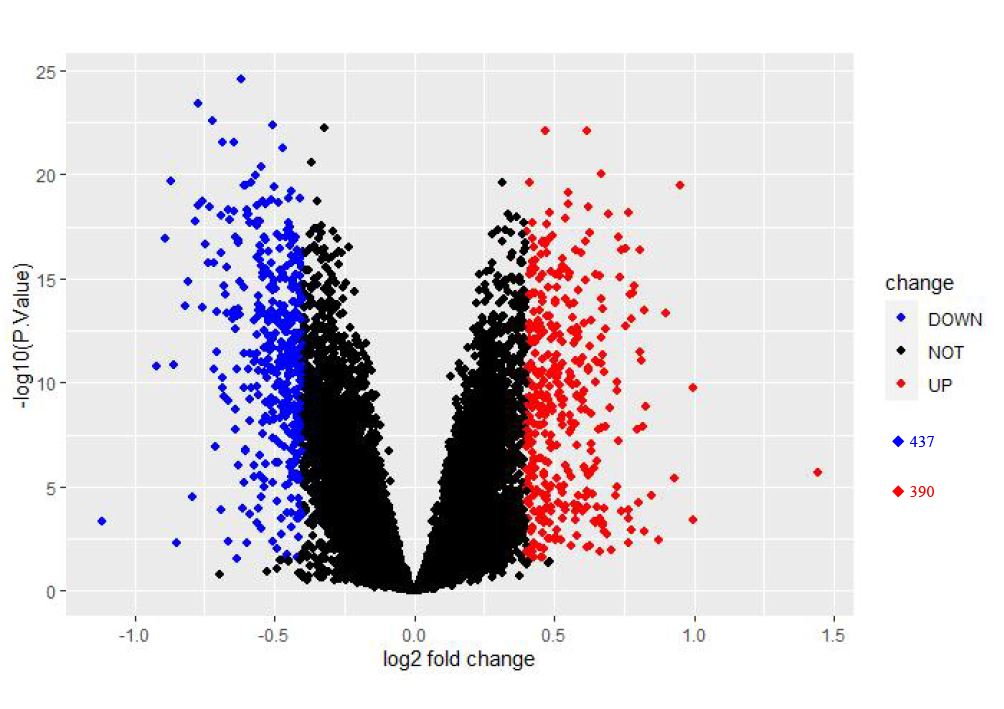

Supplement: Supplementary Figure 3 — Volcano plot showing DEGs in GSE57148. Blue represented low-expressed genes in COPD lung tissues, red represented high-expressed genes in COPD lung tissues, and black represented genes with no significant difference. [file Image_3.tif]
